# Evaluating the use of lamotrigine to reduce mood lability and impulsive behaviors in adults with chronic and severe eating disorders

**DOI:** 10.1007/s40519-021-01320-3

**Published:** 2022-03-17

**Authors:** Erin E. Reilly, Laura A. Berner, Mary Ellen Trunko, Terry Schwartz, Leslie K. Anderson, Angeline Krueger, Xinze Yu, Joanna Y. Chen, Anne Cusack, Tiffany Nakamura, Walter H. Kaye

**Affiliations:** 1 Department of Psychology, Hofstra University, Hempstead, USA; 2 Department of Psychiatry, UCSD Health Eating Disorders Center for Research and Treatment, University of California, San Diego, San Diego, CA USA; 3 Department of Psychiatry and Behavioral Sciences, University of California, CA San Francisco, USA; 4 Department of Psychiatry, Icahn School of Medicine at Mount Sinai, New York, USA; 5 Department of Psychology, Drexel University, Philadelphia, USA

**Keywords:** Bulimia nervosa, Anorexia nervosa, Emotion dysregulation, Impulsivity, Lamotrigine

## Abstract

**Background:**

Gold-standard psychological and pharmacological treatments for bulimic-spectrum eating disorders only result in remission for around 50% of patients; patients with affective lability and impulsivity represent a subgroup with particularly poor outcomes. Both dialectical behavior therapy (DBT), a treatment for emotion dysregulation, and lamotrigine, a mood stabilizer, have demonstrated promise for targeting affective lability and impulsivity; however, data exploring the combination of these interventions remain limited.

**Objective:**

We followed a group of women with recurrent dysregulated eating behaviors (*N* = 62) throughout intensive DBT treatment and compared the symptom trajectory of those prescribed lamotrigine (*n* = 28) and those who were not (*n* = 34).

**Method:**

Participants completed surveys every 2 weeks throughout treatment.

**Results:**

Group analyses suggested that all participants self-reported decreases in emotional reactivity, negative urgency, and symptoms of borderline personality disorder (BPD). The lamotrigine group reported greater elevations in BPD symptoms at baseline, but demonstrated steeper decreases in emotion and behavioral dysregulation than the non-matched comparison group. Within-subject analyses suggested that within the lamotrigine group, subjects reported greater decreases in symptoms following prescription of lamotrigine.

**Conclusions:**

Findings provide initial data suggesting that lamotrigine could be useful as an adjunctive treatment for patients with affective lability and impulsivity.

**Level of evidence:**

IV, time series without randomization.

**Supplementary Information:**

The online version contains supplementary material available at 10.1007/s40519-021-01320-3.

## Introduction

Despite progress in our understanding of the etiology and treatment of eating disorders (EDs), a significant number of patients do not achieve lasting remission of symptoms [1]. Individuals with mood lability and impulsive behaviors represent one group with a consistently poorer response to existing ED treatments [2]. For example, 25% or more of individuals with bulimia nervosa have “multi-impulsive” behaviors and are particularly prone to a chronic course of illness and high mortality. This subgroup of ED patients are defined by dysregulated eating behaviors (e.g., binge eating; self-induced vomiting) and co-occurring disinhibited behaviors and typically have high rates of psychiatric comorbidities, such as self-harm and substance use disorders [3, 4]. Consistent data suggest that impulsivity (e.g., self-injury) relates to drop-out from evidence-based treatments [5–7]. Further, patents with EDs who endorse characteristics of Cluster B personality disorders and bipolarity, including affective, identity, and behavioral instability, have worse outcomes [8–10]. Evaluation of interventions that may be effective for this specific subgroup of adults is essential to reducing the negative outcomes associated with these illnesses.

One promising treatment for multi-impulsive patients with EDs and high affective lability is dialectical behavior therapy (DBT). DBT was originally developed to target recurrent suicidal and self-harm behaviors in women with borderline personality disorder (BPD) and has been studied extensively as a treatment for behaviors associated with emotion regulation problems [11]. The DBT model teaches individuals coping strategies to facilitate effective emotional regulation [12]. DBT is considered the first-line treatment for emotion dysregulation in BPD and suicidality, and preliminary data from small-scale, open trials indicate that DBT may be effective for emotionally dysregulated ED patients [13, 14].

Though the content of DBT is well suited to the symptoms of chronic, multi-impulsive EDs, elevations in mood lability and a tendency toward rash action in the context of mood lability could interfere with engaging in treatment and learning and applying DBT skills. Indeed, data suggest that this patient population struggles to engage in other evidence-based treatments (e.g., cognitive-behavioral therapy) [15]. As such, medications that stabilize mood and reduce impulse control could serve as useful adjunct to DBT and other concurrent psychotropic medications, such as antidepressant medications. Specifically, mood stabilizers may allow dysregulated patients to better acquire and generalize skills taught in DBT. The antiepileptic drug lamotrigine, which is widely used as a mood stabilizer in bipolar disorder, has demonstrated initial success in treating patient populations characterized by labile moods and impulsivity [16, 17], although recent results from well-powered randomized controlled trials in BPD suggest it may be less efficacious [18]. With regard to its effectiveness for treating EDs, an initial case series of five patients within our program indicated that concurrent lamotrigine, often alongside an antidepressant medication, and DBT were associated with decreases in mood reactivity, impulsive behaviors, and ED behavior [19]. Results from a second open trial revealed significant decreases in impulsive and dysregulated behaviors when lamotrigine was added to DBT and concurrent selective-serotonin reuptake inhibitors (SSRIs) for nine women with EDs [20]. However, given that these investigations evaluated the simultaneous use of DBT and lamotrigine, it is unclear whether lamotrigine provides additive benefit over and above DBT.

The current study aimed to build upon existing case series research by including a comparison group of patients from the same cohort. Specifically, we compared clinical outcomes in two groups of patients with bulimic-spectrum EDs in a DBT-based program: patients who received concurrent lamotrigine treatment, and patients who were not prescribed lamotrigine. Given past work in this domain, we predicted that the addition of lamotrigine would be associated with a greater decline in affective lability and behavioral impulsivity.

## Methods

### Participants

Participants were patients enrolled in a partial hospitalization program/intensive outpatient program for EDs. To be considered for inclusion in the study, participants were required to report dysregulated eating behaviors (loss-of-control eating episodes of any size and/or compensatory behaviors) at least once per week for the past 3 months. Participants were also required to be 18–45 years old, to be female, to be medically stable, and weigh between 75 and 135% of the expected weight for their height based on the Metropolitan Life Insurance tables [21]. Patients with bipolar-spectrum, schizophrenia-spectrum, or other psychotic disorders were excluded. There were no eligibility criteria related to other medications prescribed to participants in the study.

Sixty-two patients met initial inclusion criteria for the study and were enrolled in study procedures. Over the course of treatment, 45.16% of the sample (*n* = 28) was prescribed lamotrigine, and 54.84% (*n* = 34) served as the non-matched comparison group. All participants provided written informed consent, and the local institutional review board approved this study.

## Measures

### Clinical interviews

We used the Structured Clinical Interview for DSM-5: Eating Disorders Module [22] to assess current ED diagnoses in our sample, and the MINI International Neuropsychiatric Interview [23] to assess comorbid psychiatric diagnoses. For a detailed description of the interviews, please see the Supplement.

### Biweekly self-report measurements

Participants completed a range of well-validated self-report measurements every 2 weeks over the course of treatment, including the Borderline Evaluation of Severity Over Time (BEST) [24], the Emotional Reactivity Scale (ERS) [25], the UPPS-P Negative Urgency Scale (UPPS-P) [26], and single item measures from the Eating Disorders Examination—Questionnaire (EDE-Q; [27]) to measure binge/purge frequency. For a detailed description of these biweekly assessments, please see the Supplement.

## Treatments of interest

### Dialectical behavior therapy

Each patient was enrolled in an ED partial hospitalization and intensive outpatient program. The program operates using full-model DBT and therefore includes individual sessions, skills groups, therapist consultation, and phone coaching, as outlined by Linehan [12] with adaptations described elsewhere [28]. The DBT program is administered in a partial hospitalization setting, and patients are admitted to the program if they endorse clinically significant ED symptoms that warrant admission to a higher level of care or have experienced treatment failure at a lower level of care. As described elsewhere [14, 29], ED symptoms can be incorporated readily into the DBT framework; within the current program, there were no major elements of the traditional DBT framework that were omitted, and the unique ED-specific additions to standard DBT included regular meetings with a dietician, additional groups with content from other cognitive-behavioral therapies for EDs, and medication management. Weekly individual therapy followed a DBT framework. All patients enrolled in the program begin by attending treatment for 10 h a day, 6 days a week. Pending treatment progress, patients are stepped down to 6 h a day, 5–6 days a week, followed by intensive outpatient programming (4 h a day; 3–5 days a week).

### Lamotrigine

Participants were prescribed lamotrigine by one of two staff psychiatrists or one of two psychiatric nurse practitioners. Consistent with recommendations for use of SSRIs for the treatment of both BN symptoms and co-occurring mood symptoms in EDs [30, 31], the majority of patients were also prescribed an SSRI. For lamotrigine, dosing followed a more conservative schedule than that recommended by the FDA for bipolar disorder. Specifically, study providers initiated lamotrigine dosages at 25 mg/day for ≥ 2 weeks, then increased to 50 mg/day for ≥ 2 weeks, then generally increased the dose at a rate no faster than 25 mg/day every 1–2 weeks, contingent on patient response to the medication. As such, the titration schedule varied across participants and was based on patient response, as well as staff psychiatrists’ clinical assessment. Therapeutic doses for the group ranged between 75 and 400 mg/day of lamotrigine.

### Procedures

Patients who met initial eligibility criteria were provided with information regarding the study within 1 week of partial hospitalization program (PHP) admission. Semi-structured interviews were conducted by bachelor’s- or doctoral-level research staff who were supervised weekly by two psychologists with extensive training in the administration of diagnostic interviews and attended weekly consensus meetings. Eligible participants completed biweekly assessments throughout treatment. Medication information related to changes in lamotrigine or other psychotropic medications was gathered weekly from the patient’s chart.

### Statistical analysis

We classified patients into groups based on whether they were prescribed and took lamotrigine during treatment. Initial analyses compared groups on diagnostic and self-reported variables to test their equivalence.

We examined the effect of lamotrigine on symptoms of BPD, negative urgency, emotional reactivity, binge eating, and purging using two approaches. First, we conducted multilevel models (MLMs) using the full sample to test for group x time interaction effects on symptoms. Because participants were started on lamotrigine at different points in treatment (range = 0–126 days following admission to PHP), we centered time for the lamotrigine group around the initial prescription date. “Day 0” for the non-matched comparison group was the date of admission to the clinic.

Our second set of analyses examined symptom change over time *within* the lamotrigine group (*n* = 28), excluding comparison subjects. We conducted a series of piecewise MLMs to explore changes in symptoms pre- and post-lamotrigine initiation (i.e., first day taking lamotrigine). For these analyses, time was modeled continuously and centered around the time at which participants were prescribed lamotrigine.

All MLMs were conducted using the R package *lme4* [31]. Models were fitted in a stepwise fashion, and all models reported in the current investigation represent random intercept, fixed slope models. MLMs account for data that are nested (i.e., multiple observations over time). MLMs estimate both fixed or average effects across a sample, and random effects, which model variability in effects across subjects or time points. In the current investigation, we report fixed effects, or the average effect of each variable in the model across the full sample. To handle missing data, we used full-information maximum likelihood estimation. For binge eating and purging analyses (i.e., count data), we used a negative binomial distribution, in accordance with recommendations [29].

## Results

### Baseline differences between groups

One participant provided informed consent but then dropped out of treatment during the interviewing procedures; this participant is not included in the final total for the sample and in the Tables. Frequencies of ED diagnoses, co-occurring diagnoses, and prescribed medications at baseline are available in Table 1, as well as means and standard deviations for outcome variables. These counts include all subjects consented into the study other than the participant who did not complete the interviewing procedures. On average, participants met criteria for 2–3 co-occurring diagnoses. The average length of ED illness was 11.65 years (SD = 7.69 years).

**Table 1:** Descriptive statistics, by group

DSM-5 eating disorder diagnosis	Lamotrigine	Comparison	Chi-square	*p* value
Bulimia nervosa	21	12	10.3	0.006**
Anorexia nervosa, binge-purge subtype	6	15		
Other specified feeding and eating disorder	1	7		
Comorbidity				
Major depressive disorder	19	17	2.35	0.99
Persistent depressive disorder	1	1		
Panic disorder	4	3		
Agoraphobia	1	2		
Social anxiety disorder	12	10		
Obsessive compulsive disorder	1	2		
Post-traumatic stress disorder	10	10		
Alcohol use disorder	12	9		
Generalized anxiety disorder	8	8		
Other specified anxiety disorder	0	1		
Other substance use disorder	9	8		
Class of medication prescribed at baseline				
Antidepressant	25	33	12.5	0.029*
Benzodiazepine	0	6		
Antiepileptic drug	8	4		
Antipsychotic	1	3		
ADHD medication/stimulant	5	3		
Opioid agonist	3	0		

Group comparison across true baseline			
Outcomes	Mean (SD)		Mean differences
	Lamotrigine	Comparison	
BEST	27.58 (10.95)	17.60 (8.92)	*t*(44.06) = − 3.61***
UPPS-P	38.14 (8.43)	32.85 (7.39)	*t*(40.15) = − 2.26*
ERS	55.52 (19.76)	43.23 (19.32)	*t*(42.52) = − 2.14*
WCCL	1.59 (0.45)	1.72 (0.48)	*t*(44.36) = 0.95
Binge frequency (2 weeks)	10.67 (17.23)	3.15 (3.65)	*t*(21.46) = − 1.96
Purge frequency (2 weeks)	11.81 (19.37)	7.12 (7.65)	*t*(25.04) = − 1.05

Group comparison across relative baseline			
Outcomes	Mean (SD)		Mean differences
	Lamotrigine	Control	
BEST	23.19 (9.63)	17.60 (8.92)	*t*(41.04) = − 2.10*
UPPS-P	37.40 (7.41)	32.85 (7.39)	*t*(29.26) = − 1.90
ERS	56.00 (20.11)	43.23 (19.32)	*t*(30.90) = − 2.03
WCCL	1.58 (0.42)	1.72 (0.48)	*t*(45.20) = 0.95
Binge frequency (2 weeks)	5.00 (5.48)	3.15 (3.65)	*t*(23.28) = − 1.96
Purge frequency (2 weeks)	8.75 (15.70)	7.12 (7.65)	*t*(19.46) = − 1.05

*ADHD* attention deficit hyperactivity disorder, *BEST* borderline evaluation of severity over time, *UPPS-P* UPPS-P Negative Urgency Subscale, *ERS* Emotion Reactivity Scale, *WCCL* Ways of Coping Checklist, Skills Use Subscale

*p < 0.05, **p < 0.01, ***p < 0.001

As participants were not prospectively randomized to treatment groups, we conducted two series of comparisons across treatment groups. Comparison of mean differences across groups in variables collected at intake to treatment indicate significant differences in several domains, including emotion reactivity, negative urgency, and symptoms of borderline personality disorder, such that individuals prescribed lamotrigine had higher scores in all domains (Table 1). In addition to exploring baseline differences across groups, we compared baseline assessment of the non-matched comparison group with assessments taken directly before prescription of lamotrigine in the medication group, as these time points reflected our operationalization of “baseline,” in longitudinal analyses. Results from these comparisons reveal smaller group differences only in BEST scores.

### Longitudinal group differences

The number of assessments completed by participants ranged from 0 to 20 (mean = 7.13; SD = 3.71). In total, seven participants left treatment against clinical advice prior to discharge, and 15 biweekly assessments (3.39% of total assessments) were missing due to patient absence from the program or declination to complete. We used all available data within each model, including the data of those participants with missing time points and/or those who dropped out of treatment.

**Fig. 1:**
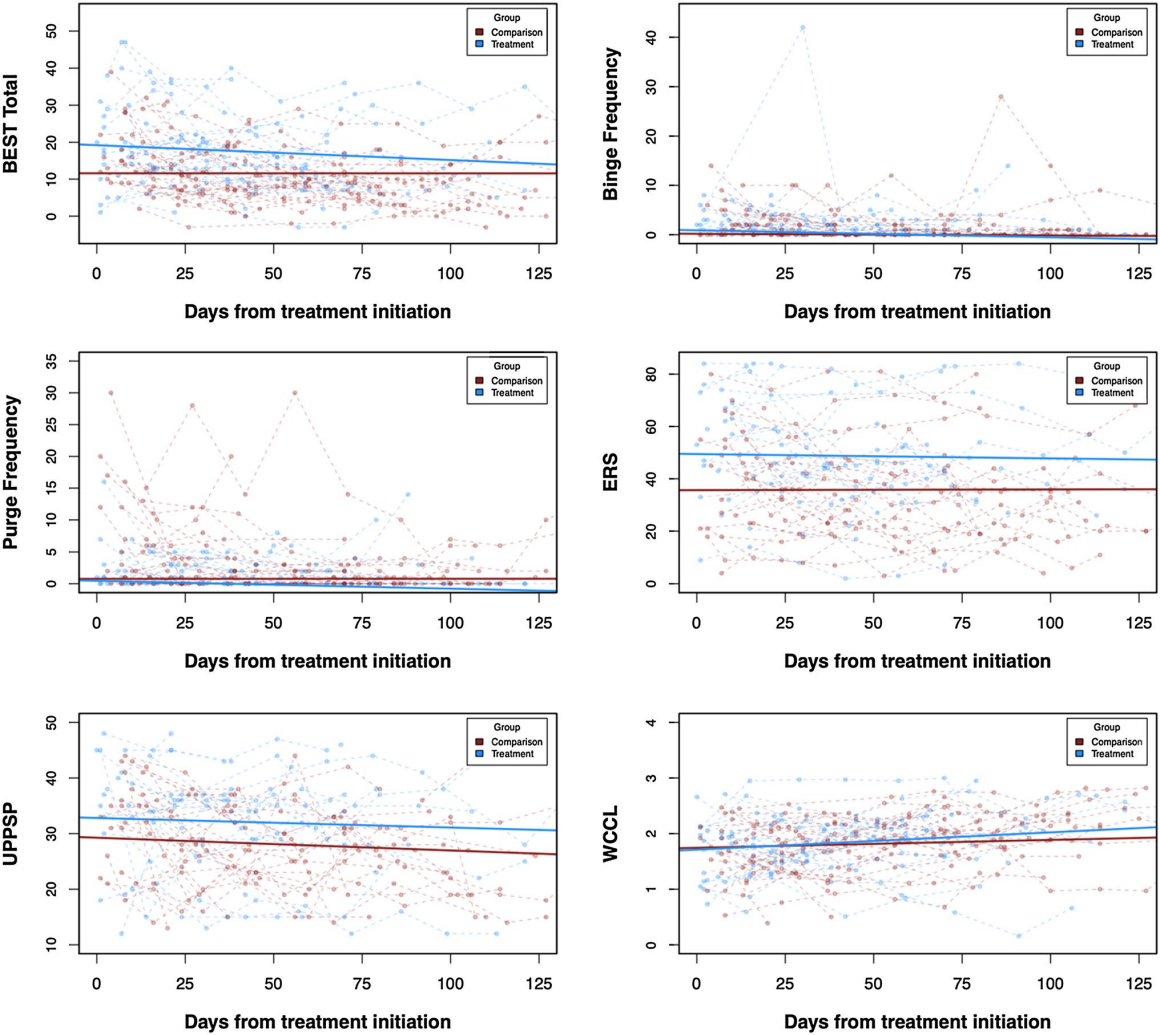
Outcome trends over time by treatment groups. Across models, the DBT + lamotrigine group is portrayed in blue, and the DBT group (comparison group) is portrayed in red

Results from each MLM are presented in Table 2 and in Fig. 1. All models indicated statistically significant main effects of time, suggesting that on average, all patients showed improvements on all measures over the course of treatment. Additional main effects and interactions are reported below.

**Table 2:** Linear mixed-effects model results for outcome variables by treatment groups

Normal distribution models (continuous outcomes)					
	Estimate	SE	*df*	*t* value	*p* value
DV: borderline evaluation of severity over time (intercept)	13.888	1.495	69.552	9.29	< 0.001***
Time	− 0.04	0.009	310.545	− 4.5	< 0.001***
Treatment	6.581	2.246	72.048	2.93	0.005**
Time × treatment	− 0.039	0.018	310.662	− 2.18	0.030*
DV: Emotion Reactivity Scale (intercept)	42.965	3.668	56.616	11.71	< 0.001***
Time	− 0.088	0.018	243.229	− 4.91	< 0.001***
Treatment	10.05	5.495	57.96	1.83	0.073
Time × treatment	0.019	0.034	239.312	0.57	0.570
DV: UPPS-P Negative Urgency Scale (intercept)	31.937	1.548	65.354	20.63	< 0.001***
Time	− 0.059	0.011	263.609	− 5.44	< 0.001***
Treatment	1.926	2.319	67.109	0.83	0.409
Time × treatment	0.051	0.02	253.500	2.62	0.009**
DV: DBT ways of coping checklist—skills use (intercept)	1.7	0.084	73.093	20.36	< 0.001***
Time	0.002	0.0005	305.470	3.06	0.0024**
Treatment	0.015	0.126	75.677	0.12	0.908
Time × treatment	0.00009	0.001	305.505	0.09	0.926

Negative binomial models (count outcomes)				
	Estimate	SE	*t* value	*p* value
DV: binge frequency (intercept)	0.299	0.295	1.01	0.311
Time	− 0.007	0.003	− 2.75	0.006**
Treatment	0.452	0.449	1.01	0.314
Time × treatment	− 0.003	0.005	− 0.5	0.620
DV: purge frequency (intercept)	1.138	0.274	4.15	< 0.001***
Time	− 0.009	0.002	− 4.54	< 0.001***
Treatment	− 0.828	0.44	− 1.88	0.060
Time × treatment	− 0.0003	0.005	− 0.07	0.950

Estimates represent fixed effects of time, treatment, and time × treatment

*DV* dependent variable

*p < 0.05, **p < 0.01, ***p < 0.001

#### BPD symptoms

Results indicated a significant effect of group and a significant interaction between time and group on BEST scores. Inspection of these effects suggested that the lamotrigine group demonstrated average BEST scores that were consistently higher than those of the non-matched comparison group, but that the lamotrigine group demonstrated steeper mean decreases in BEST scores over time compared to non-matched comparison patients (Fig. 1).

#### Negative urgency

We detected a significant group x time interaction on UPPS-P scores. The directionality of these effects suggested that on average, all participants self-reported decreases in negative urgency over the course of treatment, and the slope of this effect was greater among the non-matched comparison group.

#### Emotional reactivity, DBT skills use, and bulimic symptoms

Other than time, no effects on ERS scores, WCCL scores, binge eating, or purging were statistically significant.

### Within-group analyses (Table 3; Fig. 2)

**Table 3:** Piecewise linear mixed-effect model results for outcome variables within the lamotrigine group (grouped by pre- and post-lamotrigine)

Normal distribution models (continuous outcomes)					
	Estimate	SE	*df*	*t* value	*p* value
DV: borderline evaluation of severity over time (intercept)	20.899	1.812	33.245	11.54	< 0.001***
Pre	− 0.116	0.03	183.392	− 3.82	< 0.001***
Post	− 0.089	0.017	176.713	− 5.26	< 0.001***
DV: Emotion Reactivity Scale (intercept)	53.191	3.935	26.142	13.52	< 0.001***
Pre	− 0.023	0.057	126.093	− 0.39	0.694
Post	− 0.071	0.027	126.487	− 2.65	0.009**
DV: UPPS-P Impulsivity Scale (intercept)	34.79	1.682	31.192	20.68	< 0.001***
Pre	− 0.064	0.035	137.496	− 1.81	0.073
Post	− 0.032	0.016	136.081	− 2.04	0.043*
DV: DBT Ways of Coping Checklist—Skills Use Subscale (intercept)	1.65	0.083	38.255	19.9	< 0.001***
Pre	− 0.0005	0.002	177.867	− 0.3	0.764
Post	0.003	0.0009	171.915	3.3	0.001**

Negative binomial models (count outcomes)				
	Estimate	SE	*t* value	*p* value
DV: binge frequency (intercept)	1.04	0.272	3.82	< 0.001***
Pre	− 0.008	0.007	− 1.27	0.204
Post	− 0.015	0.004	− 3.93	< 0.001***
DV: purge frequency (intercept)	0.973	0.378	2.57	0.01*
Pre	− 0.002	0.011	− 0.15	0.880
Post	− 0.021	0.005	− 4.23	< 0.001***

Estimate indicates the fixed effect for time at pre- and post-prescription time points

*p < 0.05, **p < 0.01, ***p < 0.001

**Fig. 2:**
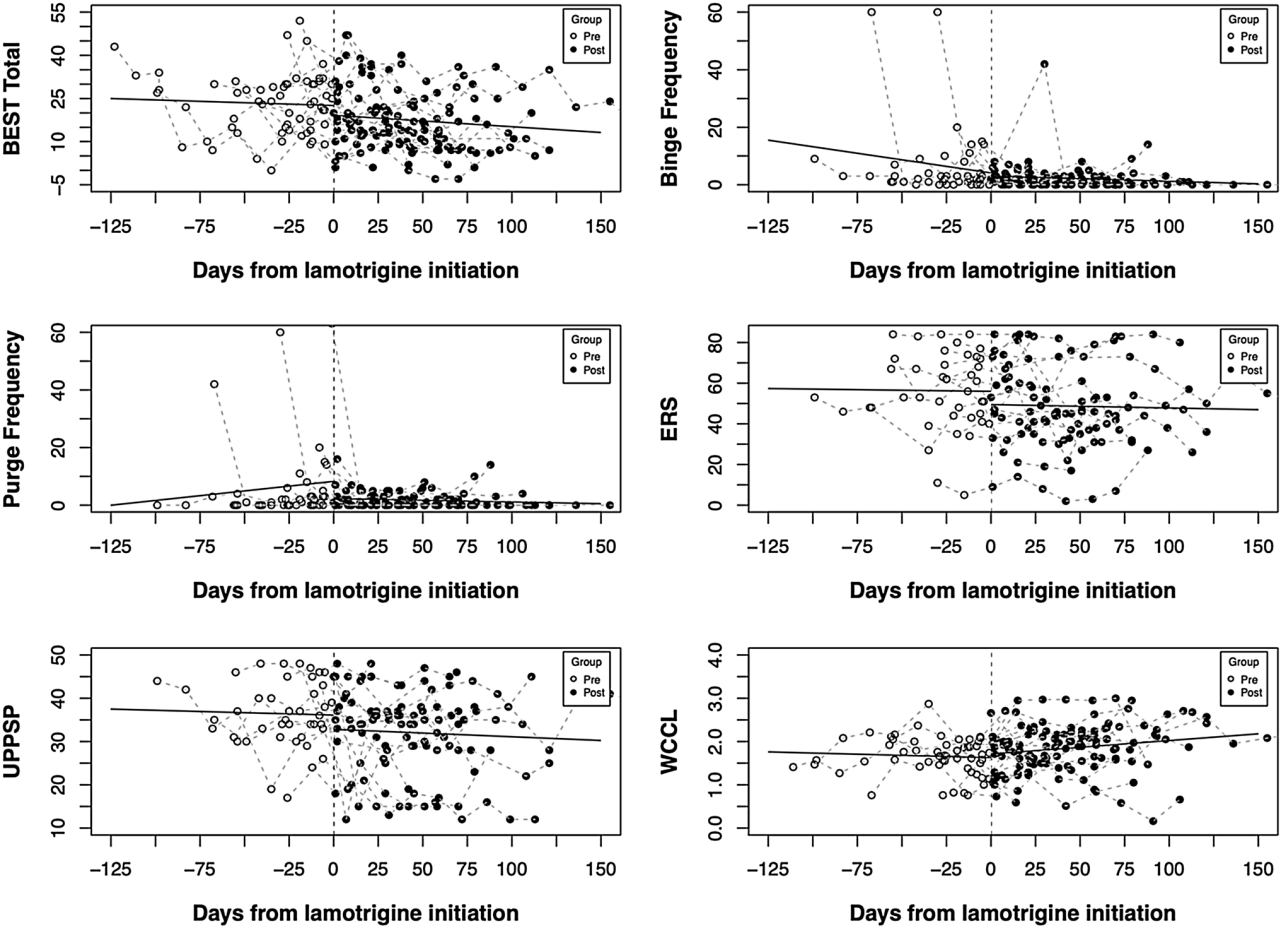
Outcome trends in pre- and post-lamotrigine initiation for the lamotrigine group. Across models, the date the individual began taking lamotrigine is depicted by the vertical dotted line; data points pre-lamotrigine are represented in white, and data points post-lamotrigine are represented in black

BEST scores decreased significantly both before and after lamotrigine initiation. This effect increased in size following the prescription of lamotrigine. Binge eating, purging, and scores on the UPPS-P, ERS, and WCCL all demonstrated a similar pattern; however, for these measures, there was no significant change in symptoms or change in DBT skills use before lamotrigine, but a significant decrease in symptoms and increase in DBT skills use after lamotrigine.

## Discussion

ED patients with multi-impulsive behaviors and BPD features such as affective lability often show poor treatment response and high rates of relapse [10]. The current study tested a mood stabilizer, lamotrigine, as an adjunctive intervention to DBT for this treatment-refractory population. This is the first study to build on an initial case series and open trial of lamotrigine for EDs by including a non-matched comparison group. Despite many limitations of the quasi-experimental study design, results provide some support for the usefulness of lamotrigine in combination with DBT for the treatment of ED patients with high affective lability and behavioral impulsivity.

All patients reported decreases over time in symptoms of BPD, affective lability, negative urgency, binge eating, and purging, and increases in adaptive skill use. However, patients prescribed lamotrigine showed a steeper decrease in BPD symptoms than those who did not. While baseline differences between groups on BPD symptoms make this interaction effect challenging to interpret definitively, and it is possible these differences simply represent regression to the mean, our findings are broadly consistent with observations that lamotrigine is helpful in decreasing impulsive behaviors associated with BPD [17, 32]. The lack of effect of lamotrigine on affective lability as operationalized by the ERS is inconsistent with several studies suggesting that lamotrigine could influence the intensity and lability of emotions in populations with BPD [17, 33], although null results for the medication’s effectiveness in targeting affective lability are not unprecedented [18]. Further, our results suggested greater decreases in negative urgency in the non-matched comparison group compared to the lamotrigine group. However, visual inspection of these differences (Fig. 1) suggested this difference was slight in nature.

Within the subgroup of patients who received lamotrigine, emotional reactivity, negative urgency, binge eating, purging, and skills use changed at a significant rate only after the initiation of lamotrigine. These analyses are limited by the nature of the data collection procedures (e.g., variability in the time frame before and after prescription); however, they do provide some preliminary evidence that that in a group of patients who reports significant affective lability and impulsivity, prescription of lamotrigine is associated with a significant decrease in self-reported emotional reactivity and impulsivity. Of note, these patients did not report significant changes in skills use, emotional reactivity, and impulsivity prior to taking lamotrigine despite being enrolled in the DBT program, consistent with the clinical prediction that lamotrigine may enable patients to better engage with and benefit from the treatment [19, 20].

The mechanism of action of lamotrigine is not yet clear; however, data from functional neuroimaging studies suggest that it primarily targets prefrontal cortical areas integral to cognitive and behavioral control. Acute doses increase resting-state connectivity among prefrontal regions [34], and after 12 weeks, lamotrigine increases activation in prefrontal regions when individuals with bipolar disorder view sad faces [35]. Longitudinal neuroimaging research paired with randomized, controlled trials are needed; however, existing findings suggest that lamotrigine’s therapeutic effects in our population may have been related to increases in prefrontal activation and connectivity that could improve the ability to control one’s behavior.

Notably, our findings are somewhat inconsistent with a recent large, randomized trial of lamotrigine for the treatment of BPD, which suggested no significant benefits of lamotrigine for BPD or depressive symptoms [18]. There are several potential explanations for the inconsistency. It is certainly possible that the methodological limitations of our study (e.g., lack of randomization; differences in groups)—detailed below—can account for our observed effects. It is also possible that other methodological differences across these studies accounts for the inconsistency. For instance, although participants in the Crawford et al. study [18] were able to enroll in other types of treatment during the trial, the rates of psychotherapy use and receipt of evidence-based therapy were not reported; therefore, it is possible that positive effects of lamotrigine only occur when administered in conjunction with evidence-based psychological treatments for BPD. Further, Crawford et al. [18] used different outcome measurements than we employed in our study and recruited individuals with complex, full-threshold BPD, limiting our ability to make direct comparisons. Altogether, while it is challenging to draw parallels between our results and that of the recent randomized controlled trial in BPD [18], the null results that emerged in this prior trial highlight the need for future rigorous attempts to replicate our findings in a large sample of individuals with dysregulated eating behaviors.

Several study limitations should be acknowledged. First, many of the patients included in the study were prescribed various medications throughout their treatment stay in addition to lamotrigine, and this makes it difficult to isolate which medications may be contributing to symptom change. However, our psychiatric providers often follow some common prescribing patterns, particularly regarding concurrent antidepressant use. As shown in Table 1, comorbid depressive and anxiety disorders are extremely common in these patients. For this reason, most patients are continued on or started on an antidepressant at the time of admission and remain on the antidepressant throughout treatment. In patients with bulimic-spectrum disorders, it is common to titrate to high-dose selective-serotonin reuptake inhibitors [30, 31], so an initial step is often dose maximization of an antidepressant. If this does not significantly improve all target symptoms, lamotrigine may be added. For patients who enter the program already having limited response to high-dose antidepressants, lamotrigine may be added at or shortly after admission.

Second, the nature of the population presented some challenges for self-report data collection. Given participants’ emotional lability, self-report data may reflect mood in any given moment and may not be an accurate depiction of the mean or variance of an individual’s mood state over a 2-week period. Furthermore, the affective instability and impulsivity of the group presented challenges to consistent data collection, as participants intermittently declined to complete assessments or took unexcused absences from treatment, where assessments were administered. Third, as noted previously, there was considerable variance in the initiation timing and dosing trajectory of lamotrigine across individuals in the study, as well as variance in the length of treatment, which makes the trajectory of treatment outcome scores difficult to interpret. Related to this limitation, we did not assess plasma or serum concentrations of lamotrigine and thus only measured adherence using patient self-report. Fourth, group assignment was not randomized, which perhaps represents the greatest limitation to our study. While our findings cannot be taken as definitive evidence of beneficial effects of lamotrigine in this population, the inclusion of a non-matched comparison group builds on promising initial evidence from open trials, supporting the assertion that future rigorous work in this domain is warranted.

The current study also had various strengths. First, relative to existing treatment studies of this severely dysregulated population, the current study includes a large sample size. Second, although a small group of patients had short treatment stays after violating the terms of behavioral contracts in treatment or discharging to higher levels of care, the mean length of stay across both groups was 72 days. The extended nature of treatment and frequent assessments provided us with numerous data points for most participants and a comprehensive picture of symptoms over the course of treatment. Third, the use of semi-structured interviews ensures accurate diagnoses across all individuals in the sample.

Our findings from a quasi-experimental design provide a number of important avenues for future research. Results support larger, randomized controlled trials to examine the effects of lamotrigine with and without DBT in patients with EDs and severe affective and behavioral dysregulation. Investigation of lamotrigine’s effects using alternative measurements, such as behavioral tasks, would also represent an important future direction. Additionally, future studies should explore whether the combination of lamotrigine with DBT is more effective in treating ED patients with full-threshold BPD.

## Conclusions

Considering data suggesting poor outcomes for individuals who struggle with chronic, impulsive ED symptoms and affective dysregulation, identifying potential treatments that can serve as an adjunct or alternative to existing interventions is a critical endeavor. In the current study, we explored the effect of lamotrigine as an adjunctive treatment to intensive DBT for EDs in individuals who struggled with impulsivity and affective lability. Despite limitations of the study design, our results provide tentative support for the use of lamotrigine in patients with binge-purge EDs, concurrent BPD features, and high levels of emotion dysregulation. Moving forward, future work should make use of randomized designs and matched control groups to pursue more definitive tests of lamotrigine as a promising treatment.

### Strength and limits

Limitations of the study include lack of randomization, resulting in unequal groups, as well as the naturalistic design of the study. Strengths include the focus on a generally understudied group in ED treatment research, the longitudinal design, and a relatively large sample compared to other work in this domain.

### What is already known on this subject?

Currently, there are very few effective treatments for individuals with multi-impulsive, bulimic-spectrum disorders, particularly those who have experienced prior treatment failure. Several case series have indicated some effectiveness for lamotrigine in the treatment of bulimic-spectrum disorders.

### What this study adds?

This study provides initial pilot support for the fact that the prescription of lamotrigine is associated with clinical benefit; randomized controlled trials represent the next step in this line of work.

## Supplementary Information

Below is the link to the electronic supplementary material.

Supplementary file1 (DOCX 17 KB)

## Data Availability

The data supporting this investigation will be made available upon reasonable request to the corresponding author.

## Code availability

The code supporting the analyses in the current manuscript will be made available upon reasonable request to the corresponding author.
