# Supplementary material for: Evaluating the use of lamotrigine to reduce mood lability and impulsive behaviors in adults with chronic and severe eating disorders
Source: Eat Weight Disord. 2022 Mar 17;27(5):1775–85. doi: 10.1007/s40519-021-01320-3 (PMC9123051; doi:10.1007/s40519-021-01320-3)
Supplement: Supplementary file 1 — Supplementary file1 (DOCX 17 KB) [file 40519_2021_1320_MOESM1_ESM.docx]

**Supplemental Materials**

**Method**

**Measures**

***Structured Clinical Interview for DSM-5: Eating Disorders Module (SCID-5-RV; First et al., 2015).*** The SCID-5-RV is a well-validated, semi-structured interview that assesses diagnostic criteria as outlined in the Diagnostic and Statistical Manual for Mental Disorders. In the current study, we used the ED module of the SCID-5 to assess current ED diagnoses in our sample.

***MINI International Neuropsychiatric Interview (MINI Version 7.0) [23].*** The MINI is a semi-structured interview that assesses mental disorders using DSM-5 diagnostic criteria. In the current study, we used the MINI to assess for psychiatric diagnoses other than EDs.

***Borderline Evaluation of Severity Over Time (BEST) [24].*** We used the BEST as a continuous measure of BPD symptoms. The BEST is a 15-item self-report scale that asks participants to report on the level of distress associated with symptoms of BPD, including affective lability, impulsive actions, problems with identity, chronic feelings of emptiness, and self-injurious behaviors. The measure has three subscales: a negative behaviors subscale, positive behaviors subscale, and thoughts and feelings subscale. Research has supported the validity and reliability of the measurement [24]. Internal consistency in our sample was acceptable (Cronbach’s α = 0.81).

***Emotional Reactivity Scale (ERS) [25].*** We used the 21-item ERS scale to measure emotional reactivity. The scale was originally developed to gauge emotional lability in samples of individuals engaging in recurrent non-suicidal self-injury and has previously demonstrated good psychometric properties. In our sample, Cronbach’s α was 0.97.

***UPPS-P Negative Urgency Scale (UPPS-P) [26].*** To assess negative urgency, or the tendency to act impulsively or rashly in response to negative affect, we used the UPPS-P Negative Urgency subscale. The scale has 12 items and demonstrated adequate internal consistency in our sample (Cronbach’s α = 0.91).

***Binge Eating and Purging Behaviors.*** We assessed binge eating and purging behaviors using single item measures from the Eating Disorders Examination—Questionnaire (EDE-Q; Fairburn, 2008). The EDE-Q is a well-validated assessment of eating pathology that asks participants to report on the frequency of specific ED cognitions and behaviors over the past 28 days. Given our biweekly assessment schedule, we asked participants to report on binge eating, self-induced vomiting, and laxative use over the previous 14 days instead of 28 days. We added together items 16 and 17, which measure self-induced vomiting and laxative use, respectively, to generate a composite measurement of purging.
